# Assessment of acute kidney injury risk using a machine-learning guided generalized structural equation model: a cohort study

**DOI:** 10.1186/s12882-021-02238-9

**Published:** 2021-02-22

**Authors:** Wen En Joseph Wong, Siew Pang Chan, Juin Keith Yong, Yen Yu Sherlyn Tham, Jie Rui Gerald Lim, Ming Ann Sim, Chai Rick Soh, Lian Kah Ti, Tsong Huey Sophia Chew

**Affiliations:** 1 Yong Loo Lin School of Medicine, National University of Singapore, 1E Kent Ridge Road, Singapore, 119228 Singapore; 2 College of Science, Health & Engineering, La Trobe University, Bundoora Campus, Victoria, VIC 3086 Australia; 3 Department of Anaesthesia, National University Health System, 5 Lower Kent Ridge Road, Singapore, 119074 Singapore; 4 Department of Anaesthesiology, Singapore General Hospital, 20 College Road, Singapore, 169856 Singapore; 5 Department of Cardiovascular and Metabolic Disorders, Duke-National University of Singapore Graduate Medical School, 8 College Road, Singapore, 169857 Singapore

**Keywords:** Critically ill, Surgery, Mortality, Acute kidney injury, Hemoglobin

## Abstract

**Background:**

Acute kidney injury is common in the surgical intensive care unit (ICU). It is associated with poor patient outcomes and high healthcare resource usage. This study’s primary objective is to help identify which ICU patients are at high risk for acute kidney injury. Its secondary objective is to examine the effect of acute kidney injury on a patient’s prognosis during and after the ICU admission.

**Methods:**

A retrospective cohort of patients admitted to a Singaporean surgical ICU between 2015 to 2017 was collated. Patients undergoing chronic dialysis were excluded. The outcomes were occurrence of ICU acute kidney injury, hospital mortality and one-year mortality. Predictors were identified using decision tree algorithms. Confirmatory analysis was performed using a generalized structural equation model.

**Results:**

A total of 201/940 (21.4%) patients suffered acute kidney injury in the ICU. Low ICU haemoglobin levels, low ICU bicarbonate levels, ICU sepsis, low pre-ICU estimated glomerular filtration rate (eGFR) and congestive heart failure was associated with the occurrence of ICU acute kidney injury. Acute kidney injury, together with old age (> 70 years), and low pre-ICU eGFR, was associated with hospital mortality, and one-year mortality. ICU haemoglobin level was discretized into 3 risk categories for acute kidney injury: high risk (haemoglobin ≤9.7 g/dL), moderate risk (haemoglobin between 9.8–12 g/dL), and low risk (haemoglobin > 12 g/dL).

**Conclusion:**

The occurrence of acute kidney injury is common in the surgical ICU. It is associated with a higher risk for hospital and one-year mortality. These results, in particular the identified haemoglobin thresholds, are relevant for stratifying a patient’s acute kidney injury risk.

**Supplementary Information:**

The online version contains supplementary material available at 10.1186/s12882-021-02238-9.

## Background

Acute kidney injury (AKI) is common within surgical intensive care units, affecting 22 to 65% of patients. Moreover, it is associated with a higher mortality risk, lengthened intensive care unit (ICU) stay, unfavorable long-term outcomes and high healthcare resource usage [1–3]. Thus, it is important to identify patients at high risk of developing AKI, so as to initiate early intervention to prevent this complication and the associated adverse outcomes.

The risk factors and incidence of AKI can vary considerably, depending on the nature of the ICU population. Previous studies have identified individual risk factors such as age, diabetes, emergency admission, major surgery and sepsis to be independent predictors of AKI [4, 5]. However, the combined effect of multiple risk factors on the risk of AKI remains unclear. To shed light on this issue, a machine learning guided generalized structural equation model was used to identify risk factors for acute kidney injury. This involved the use of decision trees which have brought more clarity to medical research in recent years [6].

The primary objective of this study was to identify patients at high risk of developing AKI in the surgical ICU population. The secondary objective was to identify the impact of AKI on both hospital and one-year mortality.

## Methods

### Patient population and study design

This was a single-centre retrospective cohort study involving all adult patients who were admitted to the surgical ICU of Singapore General Hospital between 2015 to 2017. Patients undergoing chronic haemodialysis/peritoneal dialyis prior to admission, and those admitted to a separate ICU after cardiac surgery were excluded. This study was approved by the SingHealth centralised institutional review board (Reference Number 2016/2138), with the requirement for informed consent waived for the review of clinical records.

### Data management

The collated data includes age, gender, ethnicity, body mass index, co-morbidities, baseline serum creatinine levels, ICU serum creatinine levels, pre-ICU and within-ICU haemoglobin levels, vital signs in the ICU (including mean arterial pressure and heart rate), bicarbonate levels, use of blood transfusions, type of surgery, and the presence of sepsis. The occurrence of AKI, hospital mortality, and mortality within 1 year of ICU entry was also recorded.

According to the Acute Kidney Injury Network (AKIN) criteria [7], AKI is defined as a rise in serum creatinine by ≥26.4 μmol/L or a 50% or greater increase in serum creatinine within a 48 h interval. Baseline levels of serum creatinine were determined using a previously recommended method [6]: The lowest creatinine level prior to ICU entry was selected. This baseline creatinine level had to be within 90 days prior to ICU entry. This was compared with the highest serum creatinine within the first 48 h of ICU entry. A diagnosis of AKI was made if the calculated change in serum creatinine fulfilled the AKIN criteria during this initial period. Urine output was not used to diagnose AKI in our study. Hospital mortality was defined as any mortality from ICU entry to discharge from the hospital. One year mortality was defined as any mortality within 1 year of ICU entry, and was inclusive of hospital mortality.

Haemoglobin levels recorded included the most recent haemoglobin level prior to ICU entry and the lowest haemoglobin level within the first 48 h of ICU stay. ICU bicarbonate levels recorded included the lowest level within the 1st 24 h of ICU stay. Mean arterial pressure (MAP) recorded was categorised based on the MAP categories of the Acute Physiology and Chronic Health Evaluation II score [8]. Blood transfusion was defined as any blood transfusion within a 96-h time period, 48 h before to 48 h after ICU admission. Sepsis was defined as systemic inflammatory response syndrome and the presence of infection [9]. Baseline levels of serum creatinine (as defined earlier) were used to calculate pre-ICU estimated glomerular filtration rate (eGFR). The Chronic Kidney Disease Epidemiology equation [10] was used to compute the eGFR of patients. Our study assessed pre-ICU eGFR, rather than chronic kidney disease status, as the incidence of undiagnosed chronic kidney disease in our population was expected to be high [11, 12].

### Data analysis

Exploratory analyses were performed using the Chi-square test, Fisher’s exact test, Mann-Whitney test, independent t-test and the Kaplan-Meier curve. Variables which satisfied the pre-determined criterion of *p* < 0.10 were included in the subsequent model selection. Two decision trees, namely Chi-Square Automatic Interaction Detector (CHAID) [6] and Classification & Regression Tree (CART) [13] were applied to ascertain the importance of these predictors impact on the occurrence of AKI, and to identify the optimal cut-off(s) of the quantitative predictors. Unlike CART, CHAID is able to identify multiple cut-offs, if they exist, for quantitative predictors. This is a desired feature of CHAID, as all bivariate-splitting decision trees, and the popular Receiver Operating Characteristic (ROC) Curve, are only able to identify one single cut-point. The cut-offs for predictors of AKI are not universally established, and multiple cut-offs may exist.

The findings of the above machine-learning algorithms could help to improve the predictive power of subsequent risk assessment of crucial post-operative outcomes. Analysis of these outcomes had to be done with a suitable statistical model for confirmatory analysis. The generalized structural equation model (gSEM) [14] was deemed to be most appropriate in view of its specific features and the nature of this study. It is a network of multiple equations which allowed the study outcomes to be sequentially arranged in a meaningful way. The occurrence of AKI, hospital mortality and one-year mortality were analysed with an underling Binomial distribution with logit, while the time to mortality with a Weibull distribution and a log link. The final model was identified with a backward elimination procedure (removal *p* ≥ 0.05). Collectively, the combination of decision trees and gSEM could unearth any hidden interactions among predictors, and provide a more comprehensive picture about how they were associated with the outcomes.

Thereafter, the final gSEM model’s external validity and predictive accuracy were ascertained with a 5-fold cross validation. Auxiliary models were built with the training sub-samples and their properties examined with the validation sub-samples. The predictive accuracies of the models were ascertained with the area under receiver operating characteristics curve (AUROC).

The proposed analyses were found to be adequate in terms of statistical power (> 95%) given the modelling framework of gSEM, overall sample size, number of predictors and the selected level of significance [15].

Analysis was performed using SPSS Statistics v20.0, SPSS Modeler v18 (IBM Co., Illinois, USA) and Stata MP v16 (Stata Corp, Texas, USA). All statistical tests were conducted at 5% level of significance.

## Results

A total of 1045 first-visit patients were admitted to the surgical ICU between January 2015 to January 2017. Eighty four patients were undergoing dialysis for pre-existing end stage renal failure and 21 patients had incomplete data. The final sample comprised 940 multi-ethnic patients (mean age: 61.3 ± 15.9 years, 56.7% males) whose records were assessed up to 1 year after ICU entry.

The occurrence of ICU AKI was 21.4%, of which 77.6% were AKIN stage 1, 11.4% were stage 2 and 11.0% were stage 3. Patients who developed AKI were generally older, diabetic, more likely to have ischemic heart disease, congestive heart failure, lower pre-ICU eGFR, low pre-ICU haemoglobin (Hb) levels, emergency surgery and require blood transfusion (Table 1). Within the ICU, patients who had low bicarbonate levels, low Hb levels, low mean arterial pressure (MAP ≤70 mmHg) and sepsis were more likely to develop AKI.

**Table 1:** Sample Characteristics

Characteristics and Demographics	All Patients (*n* = 940)	Without ICU AKI (*n* = 739)	With ICU AKI (*n* = 201)	*p*-value
Age, year	61.3 ±15.9	60.1± 16.3	65.7 ± 13.9	<0.001
Gender, male	532 (57%)	420 (57%)	112 (56%)	0.818
Ethnicity:				
Chinese	664 (71%)	521 (71%)	143 (72%)	0.012
Malay	94 (10%)	69 (9%)	25 (13%)	
Indian	75 (8%)	54 (7%)	21 (11%)	
Others	105 (11%)	94 (13%)	11 (6%)	
Body Mass Index (kg/m^2^)	24.0 ± 7.9	23.9± 8.2	24.6 ± 6.9	0.283
Diabetes Mellitus	221 (24%)	152 (21%)	69 (34%)	<0.001
Ischemic Heart Disease	92 (10%)	62 (8%)	30 (15%)	0.006
Congestive Heart Failure	29 (3%)	17 (2%)	12 (6%)	0.008
Pre-ICU eGFR (ml/min per 1.73m^2^)	78.1 ± 33.4	83.4 ± 30.8	59.2 ± 35.7	<0.001
Pre-ICU Haemoglobin levels (g/dL)	12.0 ± 2.6	12.3± 2.5	10.9 ± 2.6	<0.001
Type of surgery:				
Elective	432 (46%)	373 (51%)	59 (29%)	<0.001
Emergency	383 (41%)	286 (39%)	97 (48%)	
No surgery	125 (13%)	80 (11%)	45 (22%)	
Blood transfusion	320 (34%)	216 (29%)	104 (52%)	<0.001
Lowest ICU Haemoglobin in 1^st^ 48 hours (g/dL)	10.1 ± 2.2	10.3 ± 2.2	9.0 ± 1.7	<0.001
Lowest ICU bicarbonate in 1^st^ 24 hours (mEq/L)	20.6 ± 4.6	21.2 ± 4.2	18.4 ± 5.3	<0.001
ICU sepsis	232 (25%)	134 (18%)	98 (49%)	<0.001
Mean Arterial Pressure ≤ 70mmHg	197 (21%)	128 (17%)	69 (34%)	<0.001

Abbreviations used: *ICU* intensive care unit, *AKI* acute kidney injury, *eGFR* estimated glomerular filtration rate

**Fig. 1:**
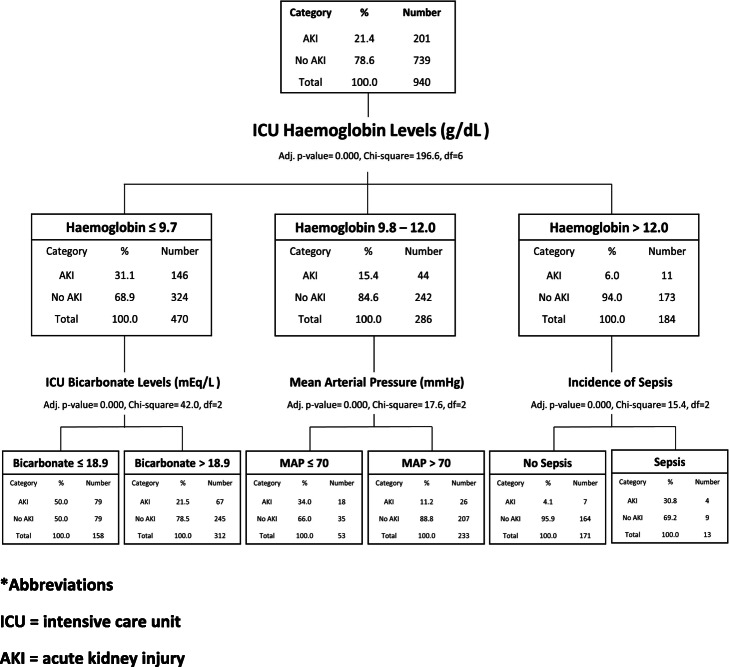
CHAID analysis of the occurrence of ICU AKI. Abbreviations used: ICU = intensive care unit. AKI = acute kidney injury

CHAID identified low haemoglobin levels, sepsis, low bicarbonate levels and low MAP as predictors for ICU AKI (Fig. 1). It identified ICU haemoglobin levels as the most important predictor for AKI, and discretized it into 3 categories (i.e., ≤ 9.7 g/dL, 9.8—12.0 g/dL and > 12 g/dL). The risk of developing AKI increased as haemoglobin levels fell.

Among those in the high-risk haemoglobin category, patients with low ICU bicarbonate (i.e., bicarbonate ≤18.9 mEq/L) had a higher chance of suffering from AKI (Fig. 1). For those in the moderate-risk category, patients with a lower MAP (≤ 70 mmHg) had a higher risk for AKI. Patients in the low-risk category were more likely to have AKI if they suffered ICU sepsis. By navigating along the branches of the CHAID decision tree one could generate the decision rules concerning the risk of AKI.

**Fig. 2:**
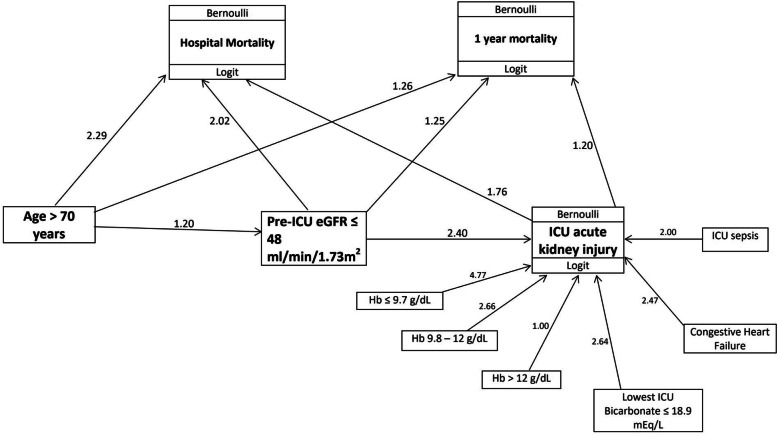
Generalized structural equation model path diagram

Further confirmatory analysis with gSEM (Fig. 2) revealed that the odds of having AKI in the ICU was significantly higher with low ICU haemoglobin levels, low ICU bicarbonate levels, ICU sepsis, low pre-ICU eGFR (defined as ≤48 ml/min/1.73m^2^, the cut-off derived via CHAID_)_ and congestive heart failure (CHF) as depicted in Table 2.

**Table 2:** Analysis of ICU AKI using the generalized structural equation model

Predictor	Adjusted Odds Ratio for ICU AKI	95% Confidence Interval
Lowest ICU Hemoglobin in 1^st^ 48 hours (g/dL)		
> 12.0	Reference	Reference
9.8 - 12.0	2.66	1.23–5.75
≤ 9.7	4.77	2.30–9.89
Lowest ICU bicarbonate in 1^st^ 24 hours (mEq/L)		
> 18.9	Reference	Reference
≤ 18.9	2.64	1.82–3.84
ICU Sepsis		
No	Reference	Reference
Yes	2.00	1.36–2.95
Pre-ICU eGFR ≤ 48 ml/min/1.73m^2^		
No	Reference	Reference
Yes	2.40	1.61–3.57
Pre-ICU congestive heart failure		
No	Reference	Reference
Yes	2.47	1.03–5.95

Abbreviations used: *ICU* intensive care unit, *AKI* acute kidney injury, *eGFR* estimated glomerular filtration rate

The mortality status was examined next. The hospital mortality rate was 18.6% (i.e., 175/940). The median time to death was 17.1 days (interquartile range: 6.3–40.4 days). AKIN stage 3 patients were at the highest risk of hospital mortality (45.5%), while stage-1 patients had a risk of 30.1%. An additional 209 deaths occurred within the first year after discharge from ICU. The total one-year mortality rate was 40.9%. The median time to one-year death was about 125 days (interquartile range: 20.6–365 days; inclusive of time in ICU).

Confirmatory analysis with gSEM (Fig. 2) identified ICU AKI to be a strong predictor of hospital mortality (AOR: 1.76, *p* = 0.004). The results were adjusted with age > 70 years and pre-ICU eGFR (Table 3). As an independent predictor, low pre-ICU eGFR had both a significant direct effect on hospital mortality, and a significant indirect effect through its impact on ICU AKI.

**Table 3:** Analysis of Hospital Mortality Status and Time-to-Death using the generalized structural equation model

Predictor	Hospital Mortality		Time-to-Death (1-Year Mortality)	
	Adjusted Odds Ratio	95% Confidence Interval	Hazard Ratio	95% Confidence Interval
Pre-ICU eGFR ≤ 48 ml/min/1.73m^2^				
No	Reference	Reference	Reference	Reference
Yes	2.02	1.36−3.00	1.25	1.06−1.49
Age (years)				
≤ 70	Reference	Reference	Reference	Reference
> 70	2.29	1.61−3.26	1.26	1.09−1.45
ICU AKI				
No	Reference	Reference	Reference	Reference
Yes	1.76	1.19−2.61	1.20	1.02−1.41

Abbreviations used: *ICU* intensive care unit, *AKI* acute kidney injury, *eGFR* estimated glomerular filtration rate

**Fig. 3:**
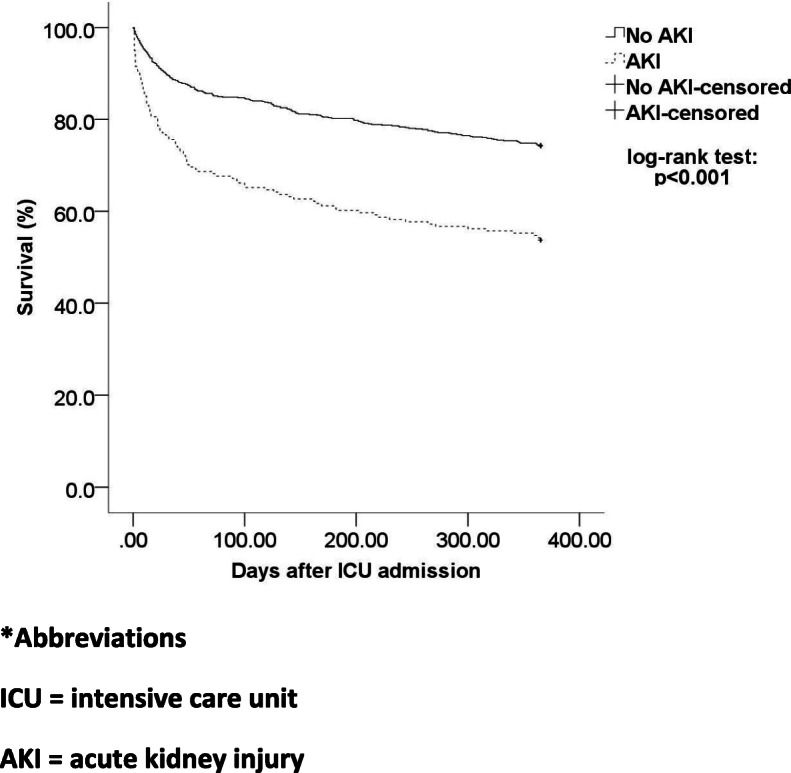
Kaplan-Meier Analysis of Time-to-Death. *Abbreviations. ICU = intensive care unit . AKI = acute kidney injury

As revealed in the Kaplan-Meier curve (Fig. 3), patients with ICU AKI had a significantly lower survival probability (*p* < 0.001). Confirmatory analysis with gSEM showed that the hazard of death was 43.3% higher with the onset of ICU AKI (Table 3). As depicted in Table 3, the predictors for occurrence of hospital mortality were also associated with time to 1-year mortality. Hence, the predictors of ICU AKI, age > 70 years, and low pre-ICU eGFR could explain both the occurrence of hospital mortality and 1 year mortality.

The results of 5-fold cross validation for the ICU AKI, hospital mortality and 1-year mortality are shown in the Additional file 1. The auxiliary models built were found to be stable and reliable, as their estimates (odds ratios, hazard ratios, 95% C.I.s and *p*-values) were numerically similar to that of the original model based on the entire sample (*n* = 940). The 95% C.I.s of the effect sizes overlapped. The AUROCs of the auxillary models for ICU AKI were 0.7537, 0.7589, 0.7950, 0.7333 and 0.7654.

## Discussion

Affecting 1 in 5 patients, AKI is common in the Singaporean surgical ICU population. The odds of hospital death is 1.76 times higher among patients with ICU AKI. It is therefore crucial to identify the potential causes of ICU AKI.

This study highlights the importance of ICU haemoglobin in stratifying AKI risk. Haemoglobin plays a key role in transporting oxygen to vital organs. Low concentrations of haemoglobin decrease arterial oxygen content, and thus oxygen delivery to the kidney. As red blood cells possess important anti-oxidative functions [16], anemia may also worsen oxidative stress in the kidneys. Thus, anemia can result in both ischemic and inflammatory organ damage, which can contribute to the occurrence of AKI [17]. The impact of anaemia has also been demonstrated in the Korean population, in which anaemia was associated with higher AKI risk and long-term mortality [18].

Two key thresholds were identified, beyond which the risk of AKI rose. When ICU haemoglobin levels were below 9.8 g/dL, the occurrence of AKI was 31.1%. This is twice as high compared with patients with haemoglobin levels between 9.8–12 g/dL and nearly 5 times higher compared to patients with haemoglobin levels above 12 g/dL. The CHAID algorithm suggested that at different haemoglobin levels, different pathologies are associated with higher AKI risk. Among those with high risk anaemia (Hb ≤ 9.7 g/dL), low bicarbonate levels have a strong association with AKI risk. Among those with moderate risk anaemia (Hb between 9.8–12.0 g/dL), MAP is strongly associated with AKI risk. Amongst those without anaemia, sepsis was associated with increased risk.

Many critically-ill patients have low bicarbonate levels. Previous studies have shown that low bicarbonate levels could predict the incidence of AKI independently [19, 20]. Our findings of how low bicarbonate levels and anaemia is associated with particularly high rates of AKI has also been shown in the rat model. Rats exposed to metabolic acidosis and induced ischemia reperfusion injury had higher levels of NF-κB and poorer glomerular filtration rate. This was in comparison to rats exposed to induced ischemia reperfusion injury alone [21]. It is postulated in literature that bicarbonate is necessary to reduce iron-mediated free radical formation, and can also help to improve oxygen delivery to the renal medulla [19, 20]. The management of low bicarbonate levels remains controversial. Its routine use in critically ill patients with metabolic acidemia has not been shown to be beneficial, and may result in deleterious side effects [22]. However, in select groups of patients, such as those at high risk of or ongoing acute kidney injury, providing bicarbonate ions via sodium bicarbonate infusion may result in improved outcomes and mortality [23, 24]. Regardless, our study highlights the importance of preventing and managing metabolic acidosis and low bicarbonate levels, particularly amongst patients with anaemia ≤9.7 g/dL, due to its strong association with complications of AKI and subsequent hospital and 1-year mortality.

In patients with haemoglobin levels of 9.8 to 12 g/dL, a low MAP ≤70 mmHg was strongly associated with AKI risk. The combination of anaemia and hypotension can compromise oxygen delivery to the kidneys. Oxygen delivery to end organs is dependent on arterial oxygen content and cardiac output [25]. Cardiac output is determined by mean arterial pressure and systemic vascular resistance. Arterial oxygen content is influenced mainly by the concentration of haemoglobin in the blood. The combination of a persistently poor mean arterial pressure and low haemoglobin concentration within the ICU may potentially overwhelm the compensatory ability of the kidney. As a result insufficient perfusion, ischemic injury and AKI can occur. Thus, in agreement with other studies, maintaining mean arterial pressure at least above 65 mmHg in patients is prudent [26], and particularly so among patients with anaemia. This study used the cut-off of 70 mmHg following the MAP risk categorization of the Acute Physiology and Chronic Health Evaluation II classification system [8]. It is not meant to weight into the debate of the exact MAP required to maintain adequate tissue perfusion.

The CHAID algorithm demonstrated the association between sepsis and AKI amongst patients with haemoglobin > 12 g/dL. This is expected, as sepsis is known to be a strong risk factor for AKI. This highlights the importance of the optimisation of sepsis management for the prevention of AKI in non-anaemic patients.

Our study had several limitations. Firstly, serum creatinine levels were not analyzed beyond the first 48 h of ICU entry. Thus patients who developed AKI at a later point in time would have been missed. The decision not to analyze beyond 48 h was made because the majority of stays in this ICU were short ones, with a median stay of 2 days. Secondly, the numbers of patients with AKI in the CHAID subgroup with hemoglobin levels > 12.0 g/dL were small, which may impact the reliability of the sepsis vs non sepsis arm. However, the model conclusion that sepsis is associated with AKI is likely sound, as it has been shown in previous studies. Thirdly, as our study population involved multidisciplinary surgical patients, its findings may not be applicable to non-surgical patients. Fourthly, our study was retrospective. Thus, predictors and outcomes may not have been assessed at the same time point or frequency in all included patients. This may result in a higher risk of misclassification bias compared to a prospective study with standardized evaluations of predictors and outcomes.

## Conclusion

AKI is a common and serious morbidity within the surgical ICU. It is associated with a higher hospital and one-year mortality risk. Both the CHAID and gSEM identified ICU haemoglobin as a key risk factor for ICU AKI. Key haemoglobin thresholds were identified, beyond which the risk of acute kidney injury rose. Furthermore, potentially synergistic risk factors for acute kidney injury were identified for each haemoglobin threshold. This is important for risk stratification and management of patients. It may also hold the key for determining the mechanism of AKI in future studies. This study is therefore the first but important step in designing patient-specific care pathways based on a patient’s specific risk profile. This is important to lower their risk for ICU AKI and subsequently their mortality risk.

## Supplementary Information

**Additional file 1.**

## Data Availability

The datasets used and/or analysed during the current study are available from the corresponding author on reasonable request.

## Abbreviations

AKI: Acute kidney injury
AKIN: Acute kidney Injury Network
AOR: Adjusted odds ratio
AUROC: Area under receiver operating characteristics curve
CART: Classification & regression tree
CHAID: Chi-square automatic interaction detector
CHF: Congestive heart failure
eGFR: Estimated glomerular filtration rate
gSEM: Generalized structural equation model
Hb: Hemoglobin
ICU: Intensive care unit
MAP: Mean arterial pressure
NF-κB: Nuclear factor kappa-light-chain-enhancer of activated B cells
