# Supplementary material for: Assessment of acute kidney injury risk using a machine-learning guided generalized structural equation model: a cohort study
Source: BMC Nephrol. 2021 Feb 22;22:63. doi: 10.1186/s12882-021-02238-9 (PMC7898752; doi:10.1186/s12882-021-02238-9)
Supplement: Supplementary file 1 — Additional file 1. [file 12882_2021_2238_MOESM1_ESM.docx]

**Appendix 1: 5-fold cross validation of ICU AKI model**

| Auxiliary model | Predictor | Adjusted Odds Ratio for ICU AKI | 95% Confidence Interval |
| --- | --- | --- | --- |
| Test  model 1 | Lowest ICU Hemoglobin in 1^st^ 48 hours (g/dL)  > 12.0  9.8 - 12.0  ≤ 9.7 | Reference  3.10  5.48 | Reference  1.23–7.79  2.27–13.22 |
|  | Lowest ICU bicarbonate in 1^st^ 24 hours (mEq/L)  > 18.9  ≤ 18.9 | Reference  2.84 | Reference  1.87–4.31 |
|  | ICU Sepsis  No  Yes | Reference  2.08 | Reference  1.34–3.23 |
|  | Pre-ICU eGFR ≤ 48 ml/min/1.73m^2^  No  Yes | Reference  2.26 | Reference  1.44–3.54 |
|  | Pre-ICU congestive heart failure  No  Yes | Reference  2.65 | Reference  0.98–7.17 |
| Test  model 2 | Lowest ICU Hemoglobin in 1^st^ 48 hours (g/dL)  > 12.0  9.8 - 12.0  ≤ 9.7 | Reference  2.16  4.06 | Reference  0.97–4.83  1.92–8.58 |
|  | Lowest ICU bicarbonate in 1^st^ 24 hours (mEq/L)  > 18.9  ≤ 18.9 | Reference  2.74 | Reference  1.81–4.16 |
|  | ICU Sepsis  No  Yes | Reference  2.23 | Reference  1.44–3.44 |
|  | Pre-ICU eGFR ≤ 48 ml/min/1.73m^2^  No  Yes | Reference  2.47 | Reference  1.57–3.88 |
|  | Pre-ICU congestive heart failure  No  Yes | Reference  2.27 | Reference  0.84–6.18 |
| Test  model 3 | Lowest ICU Hemoglobin in 1^st^ 48 hours (g/dL)  > 12.0  9.8 - 12.0  ≤ 9.7 | Reference  2.98  5.37 | Reference  1.18–7.48  2.23–12.96 |
|  | Lowest ICU bicarbonate in 1^st^ 24 hours (mEq/L)  > 18.9  ≤ 18.9 | Reference  2.41 | Reference  1.58–3.70 |
|  | ICU Sepsis  No  Yes | Reference  1.99 | Reference  1.28–3.09 |
|  | Pre-ICU eGFR ≤ 48 ml/min/1.73m^2^  No  Yes | Reference  2.33 | Reference  1.48–3.68 |
|  | Pre-ICU congestive heart failure  No  Yes | Reference  2.10 | Reference  0.84–5.25 |
| Test  model 4 | Lowest ICU Hemoglobin in 1^st^ 48 hours (g/dL)  > 12.0  9.8 - 12.0  ≤ 9.7 | Reference  2.90  4.98 | Reference  1.21–6.94  2.17–11.38 |
|  | Lowest ICU bicarbonate in 1^st^ 24 hours (mEq/L)  > 18.9  ≤ 18.9 | Reference  2.48 | Reference  1.63–3.79 |
|  | ICU Sepsis  No  Yes | Reference  2.16 | Reference  1.39–3.34 |
|  | Pre-ICU eGFR ≤ 48 ml/min/1.73m^2^  No  Yes | Reference  2.71 | Reference  1.73–4.25 |
|  | Pre-ICU congestive heart failure  No  Yes | Reference  1.66 | Reference  0.62–4.44 |
| Test  model 5 | Lowest ICU Hemoglobin in 1^st^ 48 hours (g/dL)  > 12.0  9.8 - 12.0  ≤ 9.7 | Reference  3.06  5.22 | Reference  1.13–8.23  2.01–13.53 |
|  | Lowest ICU bicarbonate in 1^st^ 24 hours (mEq/L)  > 18.9  ≤ 18.9 | Reference  2.52 | Reference  1.65–3.85 |
|  | ICU Sepsis  No  Yes | Reference  2.16 | Reference  1.38–3.37 |
|  | Pre-ICU eGFR ≤ 48 ml/min/1.73m^2^  No  Yes | Reference  2.39 | Reference  1.52–3.75 |
|  | Pre-ICU congestive heart failure  No  Yes | Reference  2.38 | Reference  0.83–6.79 |

**Abbreviations used:**

**ICU = intensive care unit**

**AKI = acute kidney injury**

**eGFR = estimated glomerular filtration rate**

**Appendix 2: 5-fold cross validation of Hospital Mortality Status and Time-to-Death**

|  | | Hospital Mortality | | Time-to-Death  (1-Year Mortality) | |
| --- | --- | --- | --- | --- | --- |
| Model | **Predictor** | **Adjusted Odds Ratio** | **95% Confidence Interval** | **Hazard Ratio** | **95% Confidence Interval** |
| Test model 1 | Pre-ICU eGFR ≤ 48 ml/min/1.73m^2^  No  Yes | Reference  1.96 | Reference  1.26−3.04 | Reference  1.26 | Reference  1.04−1.53 |
|  | Age (years)  ≤ 70  > 70 | Reference  2.35 | Reference  1.59−3.47 | Reference  1.25 | Reference  1.07−1.46 |
|  | ICU AKI  No  Yes | Reference  1.74 | Reference  1.12−2.68 | Reference  1.20 | Reference  1.00−1.44 |
| Test model 2 | Pre-ICU eGFR ≤ 48 ml/min/1.73m^2^  No  Yes | Reference  1.95 | Reference  1.24−3.05 | Reference  1.28 | Reference  1.05−1.55 |
|  | Age (years)  ≤ 70  > 70 | Reference  2.38 | Reference  1.61−3.51 | Reference  1.28 | Reference  1.10−1.50 |
|  | ICU AKI  No  Yes | Reference  1.72 | Reference  1.11−2.65 | Reference  1.15 | Reference  0.96−1.38 |
| Test model 3 | Pre-ICU eGFR ≤ 48 ml/min/1.73m^2^  No  Yes | Reference  2.01 | Reference  1.28−3.15 | Reference  1.26 | Reference  1.04−1.52 |
|  | Age (years)  ≤ 70  > 70 | Reference  2.20 | Reference  1.47−3.28 | Reference  1.25 | Reference  1.07−1.47 |
|  | ICU AKI  No  Yes | Reference  1.67 | Reference  1.07−2.62 | Reference  1.18 | Reference  0.98−1.42 |
| Test model 4 | Pre-ICU eGFR ≤ 48 ml/min/1.73m^2^  No  Yes | Reference  1.87 | Reference  1.19−2.92 | Reference  1.24 | Reference  1.02−1.50 |
|  | Age (years)  ≤ 70  > 70 | Reference  2.36 | Reference  1.59−3.52 | Reference  1.30 | Reference  1.10−1.52 |
|  | ICU AKI  No  Yes | Reference  1.77 | Reference  1.14−2.74 | Reference  1.22 | Reference  1.01−1.46 |
| Test model 5 | Pre-ICU eGFR ≤ 48 ml/min/1.73m^2^  No  Yes | Reference  2.38 | Reference  1.51−3.76 | Reference  1.27 | Reference  1.05−1.53 |
|  | Age (years)  ≤ 70  > 70 | Reference  2.52 | Reference  1.66−3.83 | Reference  1.29 | Reference  1.09−1.51 |
|  | ICU AKI  No  Yes | Reference  2.04 | Reference  1.29−3.21 | Reference  1.24 | Reference  1.03−1.49 |

**Abbreviations used:**

**ICU = intensive care unit**

**AKI = acute kidney injury**

**eGFR = estimated glomerular filtration rate**
